# Experiences of intimate partner violence (IPV) among females with same-sex partners in South Africa: what is the role of age-disparity?

**DOI:** 10.1186/s12905-024-03005-2

**Published:** 2024-03-09

**Authors:** Nicole De Wet-Billings, Brendon Billings

**Affiliations:** 1 Demography and Population Studies, Schools of Social Sciences and Public Health, Faculties of Humanities and Health Sciences, University of the Witwatersrand, Johannesburg, South Africa; 2 Anatomical Sciences, School of Anatomical Sciences, Faculty of Health Sciences, University of the Witwatersrand, Johannesburg, South Africa

**Keywords:** Intimate partner violence, Same-sex partnerships, Age-disparity, South Africa, Adjusted logistic regression, SABSSM 2017

## Abstract

**Background:**

South African women have been exposed to epidemic proportions of intimate partner violence (IPV) amongst heterosexual relationships but not much is known about same-sex partnerships. Sexual minorities are excluded from research but are subject to intimate partner violence as much as heteronormative persons. The purpose of this study is to determine the association between age-disparity and IPV outcomes among females with same-sex partners in South Africa.

**Methods:**

A cross-sectional study of the nationally representative South African National HIV Prevalence, Incidence, Behaviour and Communication Survey (SABSSM 2017) is used. A weighted sample of 63,567 female respondents identified as having a same-sex partner are analysed. IPV is measured as ever been physically and/ or sexually abused. Any experience of IPV is included in the dependent variable of this study. Descriptive and inferential statistics are used to estimate the relationship between demographic, socioeconomic, age-disparity and IPV.

**Results:**

Almost 16% of females in same-sex relationships experienced IPV and about 22% from younger partners. In female same-sex partnerships, partner age-disparity (OR: 1.30, CI: 1.18 - 1.51), type of place of residence (OR: 2.27, CI: 1.79 - 3.79), highest level of education (OR: 1.07, CI: 0.97 - 1.17), marital status (OR: 1.60, CI: 1.37 - 1.88), and race (OR: 1.47, CI: 1.41 - 1.54) are associated with an increased likelihood of violence.

**Conclusion:**

IPV programs that are specifically targeted for non-heteronormative orientations are needed. These programs should promote health equity and safety for non-confirmative sexual identities in the country.

## Background

Same-sex relationships amongst females who are cohabitating have been shown to experience higher accounts (35.4%) of intimate partner violence (IPV) during their lifetime when compared to opposite-sex cohabiting couples within the United States [1]. Despite this high prevalence of IPV, the actual cases amongst same-sex partners might actually be under-reported due to multiple factors. Research suggests that same-sex relationships are less frequently studied than heterosexual couples with regard to IPV experiences, because of the silence around violence, coupled with the stigma against same-sex partnerships [1]. A South African study, found that non-heterosexual participants were less likely to disclose their sexual orientation to healthcare workers out of fear of receiving poor care and that men are less likely to disclose compared to women [2]. Further, violence myths and misconceptions have contributed significantly to under-reporting from non-heterosexual participants. This includes the idea that women reporting abuse from same-sex partners is ‘harmless’ since women are perceived to be physically weaker or less violent than men [3]. These narratives contribute to the under-reporting of IPV in same-sex relationships and the dearth of literature on the topic.

Perhaps consequential to the reasons for under-reporting, there remains little investigation into the factors contributing to IPV in same-sex partnerships in South Africa. In heterosexual partnerships, extensive research has been done which identifies patriarchal norms and toxic masculinities [4], poverty and gender inequality [5–7] as determinants of IPV. Specifically it is known that about 13% of all women have experienced at least one form of violence and 50% of all murders of women are by an intimate partner [8, 9]. IPV is associated with increased HIV infection rates, unintended pregnancy, miscarriages, poor mental health (depression and anxiety), hypertension and others in South Africa [10–13]. However, not much is known on the levels and factors associated with IPV among same-sex couples in the country.

Partner age- difference or age-disparity, refers to a difference in age in years between intimate partners [14]. Typically measured from a single year age-difference to decades, age-disparity in heterosexual intimate relationships is common. A dual-country study in Tanzania and Uganda are among many others that found having an older male partner to be a social expectation for females [15]. The literature on female same-sex partnerships similarly show that age gaps or age-disparities are common and acceptable practices. Age-disparity is associated with several negative consequences mostly for the younger partner. For example, among adolescent and young girls and women (AYGWs) with older male partners, sexual and physical violence, the inability to negotiate safe-sex and unintended pregnancy is reported [16–19]. The width of the age-difference interval in experiences of violence is moot. One study in Nigeria found that as the age-interval widens, the experiences of IPV decrease with rates of 27.0, 23.7, 22.0 and 18.7% among couples with age differences of 0–4, 5–9, 10–14 and ≥15 years respectively [20]. While a study on spouses in India found that violence rates peaked when there was an age gap of 3-4 years (24.4%) and then declined to 21.9% if the age difference was 5 or more years [21]. Given the power imbalance in age-disparate heterosexual relationships it is worth investigating if similar trends are seen in same-sex couples and since females are disproportionally affected, it is worth researching in female same-sex relationships.

The silence around same-sex partnerships and IPV as autonomous occurrences becomes a double burden for persons with a same-sex partner preferences, which is scarcely researched in African countries. The current study aims to determine the association between age-disparity and IPV outcomes among females with same-sex partners in South Africa, a country where IPV as a form of Gender Based Violence (GBV) remains a prominent health issue. In achieving the aim of this study, the levels and types of violence experienced by females in same-sex partnerships separately are described and analysed with age-discrepancy between partners as a main predictor characteristic of violence.

## Methods

### Data source and sample size

Data are from the South African National HIV Prevalence, Incidence, Behaviour and Communication Survey, 2017 (SABSSM 2017). This nationally representative dataset collected over 39,000 (n) respondent’s HIV status, demographic, socioeconomic and behavioural characteristics [22]. For this study, only youth and adults (>=15 years old) were included. To identify same-sex partners, a question in the survey pertaining to the sex of most recent sexual partner was used and only respondents who reported their last sexual partner as the same-sex is used. Sampling weights were computed by HSRC researchers to account for unequal sampling at small areas layers, household level, and individual response to the questionnaire and HIV testing to correct for potential bias due to unequal sampling probabilities [23]. Final individual sampling weights were benchmarked to 2017 mid-year population estimates by age, race, sex, and province to provide population estimates [23]. A weighted sample (N) of 63,567 female respondents were identified as having a same-sex partner. Further, 10,100 (N) or 15.89% reported having experienced at least one form of IPV. The dataset, does not include questions pertaining to emotional violence, hence our definition of IPV in the current study only includes physical and sexual violence.

### Study Variables

The outcome of interest in this study was IPV. The questionnaire asked 11 questions pertaining to various forms of physical, and sexual violence from an intimate partner. Three IPV outcome variables were derived from this data. First, a count variable which accumulates the number of types of IPV experiences per respondent was generated and used for descriptive analysis. Second, a categorical variable that measures at least one type of physical, at least one type of sexual and both (at least one physical and one sexual) was used to describe the complexity of IPV and to highlight under-reporting of sexual IPV independent of physical IPV. Third a binary variable of any IPV experience, yes or no was generated to conduct inferential statistics on the relationship between respondents’ characteristics, including partner age difference, and IPV within same-sex partnerships.

Using the respondent’s age and the reported age of their most recent sexual partner, partner age-difference or age-disparity, the main predictor variable of the study, was created. The majority of respondents with a same-sex partner reported having an older partner (94.48%), therefore a variable of ‘same’, ‘partner younger’ or ‘partner older’ was generated.

The study also controls for several demographic characteristics of the population. These are age group, in ten-year age intervals starting at 15 years old and ending at 70 years old. Also race / population group, which represents the broad racial categories in South Africa of African/Black, Coloured, White and Indian or Asian decent and marital status where divorced and widowed respondents were grouped together and kept separate are those who are married and living together. To further explain the status of relationships, ‘living arrangements’ was added and grouped respondents as ‘living with spouse’, living without spouse’, ‘cohabiting’ (not married but with a partner), ‘not with partner’ (for the divorced or widowed respondents) and single (referring to never married and not in a relationship). The study also controlled for socioeconomic status of the respondents and here highest level of education, grouped into ‘primary’, ‘secondary’, ‘tertiary’ and ‘unknown’ were used. Second, employment status, measured as ‘employed’, ‘unemployed’ and ‘student’, and type of place of residence, grouped as ‘urban’, ‘rural’ and ‘urban informal’ (townships) were used as measures of socioeconomic status in this study.

### Data Analysis

Descriptive and inferential analysis were conducted in this study using STATA 14. Cross-tabulations showing the frequency and percentage distributions of the respondents’ characteristics and IPV experiences were investigated. An unadjusted logistic regression model was fit to the data to show the probability of IPV by partner age-disparity and other control variables. Statistical significance is set at *p*-values <0.05.

### Patent and public involvement

No patent involved.

## Results

Most of the female respondents in the study are between the ages of 25 and 34 years old (about 45%) and are never married (66.13%). Further, approximately 41% are employed and about 62% have a secondary education.

**Fig. 1:**
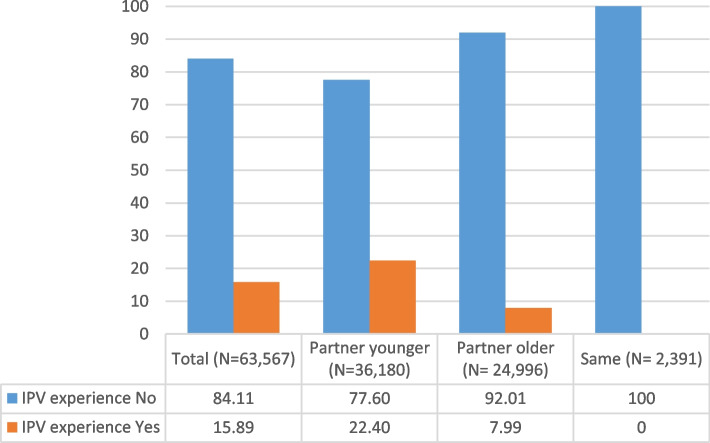
Partner age-disparity by IPV experience among female same-sex partners in South Africa

Figure 1 illustrates the distribution of IPV by age- disparity of partners. Almost 16% of females in same-sex partnerships experienced IPV. Further, 22.04% of females with a younger partner and 7.99% of those with an older partner experienced at least one type or form of IPV. No females with same-sex partners of the same age reported IPV.

Table 1 shows the weighted percentages of reporting of violence by respondents. The table shows that being pushed by an intimate partner is the most prevalent form of violence in female (21.88%) same-sex relationships. Among female same-sex partnerships slapping (19.75%), kicking (16.51%), punching (14.95%) and arm twisting (13.21%) are also highly prevalent. Sexual IPV was barely reported by females in same-sex relationships. For females with younger partners, 75.74% reported being kicked compared to 24.26% with older partners. Further, all forms of physical violence (pushing = 74.31%, slapping = 74.63%, arm twisting= 69.66% and punching=73.23%) were all higher among females with younger partners compared to those with older partners.

**Table 1:** Percentage distribution of specific form of IPV by type of same-sex partnership

Type of IPV*		Total		Younger		Older	
		Frequency (N)	%**	Frequency (N)	%	Frequency (N)	%
**Physical**	Push	7769	21.88	5773	74.31	1996	25.69
	Slap	7015	19.75	5235	74.63	1779	25.37
	Arm twist	4687	13.20	3265	69.66	1422	30.34
	Punch	5310	14.95	3888	73.23	1422	26.77
	Kick	5862	16.51	4440	75.74	1422	24.26
	Threat	606	1.71	606	100	0	0
**Sexual**	Forced sex	1422	4.00	0	0.00	1422	100
	Perform other sex act	1422	4.00	0	0.00	1422	100
	Unwanted sex	1422	4.00	0	0.00	1422	100
**Physical only**		8678	24.44	8104	93.38	574	6.62
**Both**		1422	4.00	0	0	1422	100

^*^choking, forced with threats and sexual IPV only was removed because there were no responses to this form of violence experience

^**^denotes total percentage

**Fig. 2:**
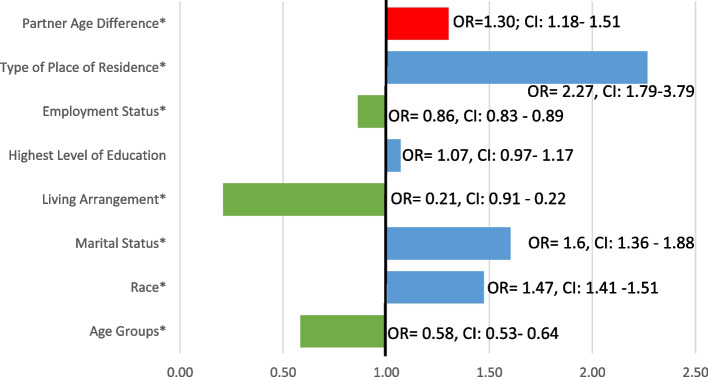
Adjusted Logistic regression results shows the odds of IPV by female respondents’ characteristics in same-sex relationships

Table 2 shows the frequency and percentage distribution of IPV experience overall and by partner age-discrepancy. Overall 15.89% of females with same-sex partners experience violence. Further, violence experiences are highest among 15-24 year olds (29.48%), White (43.64%), Coloured (*N*=217), never married (18.81%), not living with their partner (23.81%), those who did not disclose their highest level of education (26.14%), students (67.02%) and urban residents (18.01%). By partner age-discrepancy, 80.24% of IPV experience was by same-sex partners who are younger than the respondents. By younger partners, 91.30% of the respondents in the 25-34 years old age range experience IPV, 77.45% are African/Black, 90.12% are married and live with their spouses, 69.42% have a secondary education and all of those who reported violence by a younger partner are either unemployed or students, with 92.37% also being urban residents. Among the 19.76% who reported IPV by an older partner, 45.12% are in the age-group 35-55 years old, 22.55% are African/ Black, 22.50% are never married, 39.34% are cohabiting, 30.58% have a secondary education, 63.99% are employed and 70.11% live in urban informal areas. Figure 2 shows that as partner age-disparity (OR: 1.30, CI: 1.18 - 1.51) increases the probability of IPV among females in same-sex relationships. Further, the likelihood of violence by an intimate partner is higher by type of place of residence (OR: 2.27, CI: 1.79 - 3.79), highest level of education (OR: 1.07, CI: 0.97 - 1.17), marital status (OR: 1.60, CI: 1.37 - 1.88), and race (OR: 1.47, CI: 1.41 - 1.5). Employment status (OR: 0.86, CI: 0.83- 0.89), living arrangement (OR: 0.21, CI: 0.19 - 0.23) and age (OR: 0.58, CI: 0.53 - 0.64) are associated with decreased odds of IPV among same-ese female partnerships.

**Table 2:** Weighted frequency (N) and percentage distribution of IPV experience (yes/no) by respondents characteristics in female same-sex relationships

Respondents Characteristics		IPV Experience		Partner Age Disparity	
		No	Yes	Partner Younger	Partner Older
Total	N	N (%)	N (%)	N (%)	N (%)
	63 567	53 467 (84.11)	10 100 (15.89)	8 104 (80.24)	1 996 (19.76)
Age Group (*p*-value= 0.013)*					
15-24	8 896	6 273 (70.52)	2 622 (29.48)	2 622 (100)	-
25-34	28 892	24 782 (85.77)	4 110 (14.23)	3 753 (91.30)	358 (8.70)
35-55	19 103	15 952 (83.5)	3 151 (16.50)	1 729 (54.88)	1 422 (45.12)
56-70	6 677	6 460 (96.76)	217 (3.24)	-	217 (100)
Race (*p*-value= 0.023)*					
African/Black	48 389	40 499 (83.69)	7 890 (16.31)	6 111 (77.45)	1 779 (22.55)
White	2 388	1 346 (56.36)	1 042 (43.64)	1 042 (100)	-
Coloured	217	-	217 (100)	-	217 (100)
Indian/Asian	10 032	9 704 (96.73)	328 (3.27)	328 (100)	-
Marital Status (*p*-value = 0.022)*					
Married	19 681	17 488 (88.86)	2 193 (11.14)	1 976 (90.12)	217 (9.88)
Never Married	42 040	34 132 (81.19)	7 907 (18.81)	6 128 (77.50)	1 779 (22.50)
Divorced/widows	1 847	1 847 (100)	-		
Living Arrangement (*p*-value = 0.031)*					
With spouse	16 821	14 629 (86.96)	2 193 (13.04)	1 976 (90.12)	217 (9.88)
without spouse	4 153	4 153 (100)	-		
cohabiting	4 721	3 812 (80.75)	909 (19.25)	551 (60.66)	358 (39.34)
not with partner	19 601	14 933 (76.19)	4 667 (23.81)	3 246 (69.54)	1 422 (30.46)
Single**	18 271	15 940 (87.24)	2 331 (12.76)	2 331 (100)	-
Highest level of education (*p*-value= 0.019)*					
Primary	6 685	6 685 (100)	-		
Secondary	39 619	33 092 (83.53)	6 527 (16.47)	4 531 (69.42)	1 996 (30.58)
Tertiary	4 848	4 520 (93.24)	328 (6.67)	328 (100)	-
Unknown	12 416	9 171 (73.86)	3 246 (26.14)		
Employment Status (*p*-value= 0.029)*					
unemployed	33 427	29 069 (86.96)	4 359 (13.04)	4 359 (100)	-
student	3 912	1 290 (32.98)	2 622 (67.02)	2 622 (100)	-
employed	26 227	23 108 (88.11)	3 119 (11.89)	1 123 (36.01)	1 996 (63.99)
**Type of place of residence (***p*-value= 0.036)*					
Urban	41 754	34 234 (81.99)	7 521 (18.01)	6 947 (92.37)	574 (7.63)
Urban informal	18 416	16 388 (88.99)	2 028 (11.01)	606 (29.89)	1 422 (70.11)
Rural	3 397	2 845 (83.77)	551 (16.23)	551 (100)	-

^*^P-Value<0.05; **single refers to not having a defined partner

## Discussion

The purpose of this study was to investigate the relationship between age-disparity and IPV in female same-sex relationships in South Africa. The current study contributes to a dearth of research conducted on same-sex couples in the country [24–26]. Within female same-sex partnerships, there is a higher probability of experiencing violence if there is an age-difference with their partner. In same-sex couples, gendered power dynamics are downplayed, with research showing that female same-sex partners do not believe their partners could be physically abusive because violence is a masculine trait [27]. However, in same-sex relationships violence does occur among females and is increasingly being researched and observed [28–32]. Among the few studies that have been done it was found that rates of violence within same-sex female relationships are high with a lifetime prevalence rate of 43.8% [33] and the IPV that occurs within relationships is associated with social (race, socioeconomic status and age) differences [34]. One study argues that the generational differences between older and younger women in same-sex relationships who experience different levels of comfort and self-acceptance regarding their sexual orientation in general could be a source of violence [35]. Finding evidence of younger perpetrators of IPV in female same-sex relationships was difficult, the only evidence to support this result was found in a study that shows younger females who face difficulty in disclosing their sexual orientation to family and friends, experiences of intergenerational transmission of violence (from or seeing parents for example) and feelings of jealousy resulting in aggressive, combative and even self-harming behaviours [36]. Researchers have found that females in same-sex relationships do not report violence because of homophobia, fear of losing custody of their children as well as the perception that services are only available for heterosexual women [30–32].

Overall, reporting of sexual IPV in this study is low. It should be noted that sexual violence within same-sex relationships is as common if not more common as identified in heterosexual relationships [37]. Among same-sex couples studies have found that under-reporting of physical and sexual violence is also attributed to fears of the police and justice systems being homophobic and having heterosexualism attitudes [38, 39]. In general sexual violence, has been shown to be under-reported for varying reasons. Namely there is a widespread belief that that sexual violence does not occur within consensual relationships, regardless of the sexual orientation of the individual [40–42].

In this study, being ‘pushed’ is the most reported form of violence. Other research has also found a high prevalence of this type of violence with one study of self-identified gay and bisexual men showing a prevalence of 89.7% reporting being pushed by a partner [43]. Of interest, however, is that being pushed is not the only form of violence experienced, with many respondents in this study reporting more than one form of physical or sexual IPV. It was similarly found that often victims of IPV will not experience only one form of violence, but will suffer from many types [44–46] and it is well known that violence in relationships escalates in terms of frequency and severity over time [47, 48]. Often abuse starts with verbal or emotional attacks followed by more severe with incidents of physical and sexual violence and sometimes results in death [9, 49].

This study has a few strengths. First, the weighted data on females has provided sufficient cases for inferential analysis and to produce nationally representative results. Second, the quantitative study design allows for broad profiling of the factors associated with IPV among same-sex couples which can be used for more targeted, qualitative studies to understand why certain characteristics are risk factors for violence within these relationships. Finally, the study is one of very few in the country and continent that examines the harmful IPV experiences of those in same-sex relationships. Studies such as this debunks denialism around IPV among same-sex partners and highlights the need to prioritise and assist the victims in relationships.

There are however, a few limitations to this study too. First, is the possibility that same-sex relationships are under-reported in the survey. Due to the pre-existing stigma around same-sex partnerships, there is the strong possibility that many respondents did not honestly state the sex of their most recent partner. Again other studies have found that under-reporting of same-sex partnerships is common [50–52]. Second, the list of forms of violence is not exhaustive and might exclude some types of violence, including all forms of emotional abuse, or might not be understood by participants. An example of the former might be ‘burning with a lighter or other device’, ‘cutting or stabbing’, which are not on the list and an example of the latter would be ‘strangling’ which is called ‘choking’ in the current survey. Third, the accuracy of the age-disparity variable might be affected by the respondents estimating, and not knowing the actual age of their most recent sexual partner. For this reason age-disparity was categorised into three groups and not used in single years. Fourth, the data are cross-sectional and therefore causation cannot be inferred from the results. Included in this latter point of causation is the identification of the sex of the abusive partner. This study cannot control for the possibility that IPV was experienced prior to the three most recent partners and that the abusive partner might not have been by a female partner.

## Conclusions

The study reveals that partner age-disparity correlates with intimate partner violence (IPV) in female same-sex relationships, akin to patterns observed in heterosexual partnerships. Given the elevated risk of abuse in age-disparate relationships among same-sex couples, further research on IPV experiences in this demographic is imperative. Same-sex couples, marginalized and stigmatized, grapple with a dual silence surrounding their sexual orientation and violence within relationships featuring age differences. Subsequent studies on IPV in same-sex couples, particularly qualitative inquiries into partner characteristics and relationship dynamics, are warranted in South Africa and beyond. These studies should scrutinize the contributing factors behind age-disparity as they seek to comprehend and address the challenges faced by victims. Such research could aid program directors and policy-makers by identifying factors linked to IPV across diverse sexual identities and relationships, thereby mitigating the prevalence of violence in the country.Lastly, surveys and data collection tools should incorporate additional inquiries to gauge the impact of IPV in same-sex couples. This comprehensive approach will facilitate robust analyses of violence across a broader population, informing policies and programs aimed at reducing IPV in same-sex relationships.

## Data Availability

This study uses secondary data which is free to download from the South African Human Sciences Research Council website at: https://repository.hsrc.ac.za/handle/20.500.11910/15468.
